# Effect of strength training on orthostatic hypotension in Parkinson’s disease—a pilot study

**DOI:** 10.1007/s10286-022-00870-5

**Published:** 2022-06-15

**Authors:** Svenja Hüsch, Joana Schauermann, Bruno Fimm, Christina Haubrich, Kathrin Reetz, Jörg B. Schulz, Andrea Maier

**Affiliations:** 1 Department of Neurology, Medical Faculty RWTH Aachen University, Pauwelsstraße 30, 52074 Aachen, Germany; 2 JARA-BRAIN Institute Molecular Neuroscience and Neuroimaging, Forschungszentrum Jülich GmbH and RWTH Aachen University, 52074 Aachen, Germany

**Keywords:** Parkinson’s disease, Strength training, Orthostatic hypotension, Cognitive decline, Tilt table

## Introduction

Parkinson’s disease (PD) is accompanied by orthostatic hypotension (OH) and neuropsychological impairment [1, 2]. In neurogenic OH, systemic vasoconstriction or increase in heart rate during standing is reduced or absent due to impairment of the autonomic nervous system. OH therapy includes pharmacologic and nonpharmacologic approaches. Counter maneuvers and compression bandaging can counteract the lack of vasoconstriction and postural venous pooling by passively increasing vascular pressure and thus venous return [1, 2]. Lower-body strength training has been established as part of the standard treatment for patients with OH [1], but there are no evidence-based trials describing the extent of therapy and effects of strength training on OH and cognition in PD. This pilot study investigated whether 8 weeks of leg muscle strength training in addition to standard treatment improved orthostatic blood pressure decrease in PD patients with OH during tilt-table test.

## Materials and methods

This single-center, randomized, single-blinded (investigator blinded, participant unblinded), controlled preliminary intervention study was conducted with a crossover design (two treatments, three periods) [3]. OH was confirmed by a 10-min tilt-table test and implied initial (blood pressure decline of > 40 mmHg systolic/20 mmHg diastolic < 15 s after standing), classic (decline of > 20 mmHg systolic/10 mmHg diastolic within 3 min of standing), and delayed OH (decline beyond 3 min) [4]. Non-neurogenic causes of OH were excluded by history, physical examination, electrocardiogram, and laboratory tests. Inclusion criteria were a diagnosis of PD, the presence of OH, sufficient mobility to perform strength training, and a minimum score of 24 out of 30 on the Montreal Cognitive Assessment (MoCA) [5].

Of 331 PD patients screened between April 2014 and October 2018 for symptoms of OH, 29 patients (16 females, mean age 69 ± 9.6 years, disease duration of 4.3 ± 3.8 years) met all inclusion criteria. Randomization was performed by random permutations in two blocks, with 15 patients assigned to group A and 14 to group B. Five patients in group A and four patients in group B dropped out after the first or second visit. Three patients had type 2 diabetes (one dropout, one each in group A and B), four patients had clinical signs of polyneuropathy (two dropouts, one each in group A and B) as an additional risk factor for autonomic dysfunction and 17 patients suffered from arterial hypertension (seven in group A and ten in group B).

Strength training was applied to both groups in a crossover design. After the baseline visit, group A had a training phase of 8 weeks, while group B served as the control group. This was followed by visit 2, an 8-week washout period, and visit 3. Group B then received strength training, while group A served as the control group. This was followed by visit 4, the washout phase, and visit 5. Group B received another 8 weeks of strength training, while group A remained the control group. The last visit took place after a total of 40 weeks.

At each of the six clinical visits between the therapy/washout phases, patients received advice on nonpharmacological treatment of OH [1, 2] and medication dosages were adjusted to minimize their influence on OH. Pressor medications (midodrine or fludrocortisone) were used and adapted in a few patients during clinical routine treatment. Tilt-table examination and heart rate variability testing (respiratory sinus arrhythmia RSA) were performed between 8 a.m. and 10 a.m., on an empty stomach without ingestion of morning medication, nicotine, or caffeine, and without wearing compression garments. The tilt-table test was performed after 10 min in the supine position, for 10 min in an upright position of 70°, with continuous noninvasive blood pressure measurement (Fan 4.1.0, Bio Sign GmbH, Ottenhofen, Germany). “Maximal systolic blood pressure change” (difference of mean systolic blood pressure during 5 min supine and minimum systolic blood pressure upright during 10 min of standing) was chosen as primary outcome variable to quantify the absolute blood pressure drop. Artifacts were removed before analysis, so that the maximum blood pressure drop did not overestimate OH. Coprimary endpoints were “maximal diastolic change”, “mean systolic/diastolic change” (difference of mean blood pressure supine and mean blood pressure during the 10-min standing period) and RSA. The standing time in which the respective minimum and maximum values were reached was expressed as the “time to reach systolic/diastolic minimum” and the “time to reach maximal heart rate (min)”. Secondary endpoints were the test results of mobility tests, cognitive tests, questionnaires, and the transcranial Doppler.

The respective therapy group received 8 weeks of training with a frequency of two sessions per week. Each 45- to 60-min training session consisted of a 20-min warm-up program (bicycle ergometer or cross-trainer) with individually set resistance, with 20 min of strength training of the leg extensor and knee flexor muscles on the leg press, leg curl, and leg extension machines with individually adjusted weights thereafter. After three sets of ten repetitions per device, the training was completed with 20 min of balance training on unstable surfaces such as balance pads. The individual strength limit of the training day of a patient was tested by gradually adjusting the weights. It was redefined for each training day. If possible, the training intensity was increased with each session, depending on the patient's performance on that day. The patient did not have to train beyond their performance limit. Patients also performed calf muscle exercises three times per week at home during the exercise periods. Exercises were not to be performed during washout periods, see supp. Figure 1 and 2.

Statistical analyses were performed using IBM SPSS Statistics 25. The significance level for all analyses was set a priori at a type I error of α = 0.05. The study was powered for at least eight patients in each group to detect an improvement of approximately 10 mmHg (range 5–15 mmHg, power, 80%) in the primary outcome. Crossover analysis of the primary and co-primary endpoints was performed using a linear mixed model (LMM), with fixed effects for treatment and period, and random patient effects. The fixed treatment effect can be interpreted as the estimated treatment difference (ES) between study groups.

## Results

Patients had mild-to-moderate bilateral disease, and mild cognitive impairment (median MoCA value of 25.5 of 30 points). The mean “maximal systolic blood pressure decline” was approximately − 50.5 ± 22.4 mmHg (see Table 1). The most frequently reported autonomic symptoms were dizziness (80%) and syncope (40%). There was an improvement in “maximal systolic blood pressure change” (ES = 4.77 mmHg, 95% confidence interval CI from - 5.42 to 14.96) and “maximal diastolic blood pressure change” (ES = 2.46 mmHg, CI = − 3.46; 8.37), but neither of these effects nor those on any of the other coprimary endpoints were significant. Thus, we did not further analyze secondary endpoints on cognitive function, mobility, and quality of life.

**Table 1:** Baseline demographic and clinical characteristics

	Group A (*n* = 15)	Group B (*n* = 14)	Total (*N* = 29)
Age (years), mean ± SD (median)	67.7 ± 10.9 (72)	70.4 ± 8.1 (72)	69 ± 9.6 (72)
Female, *n* (%)	10 (66.7)	6 (42.9)	16 (55.2)
Hypertension, *n* (%)	7 (56.7)	10 (71.4)	17 (58.6)
Atrial fibrillation, *n* (%)	5 (33.3)	1 (7.1)	6 (20.7)
Duration of nonmotor symptoms (years), mean ± SD (median)	12.5 ± 23.3 (2)	1.6 ± 2.9 (.5)	7.2 ± 17.5 (1)
Reported autonomic symptoms at baseline visit			
Dizziness, *n* (%)	11 (73.3)	13 (92.9)	24 (82.8)
Syncope, *n* (%)	5 (33.3)	7 (50)	12 (41.4)
Upper gastrointestinal symptoms (dysphagia, nausea, vomiting), *n* (%)	2 (13.3)	2 (14.3)	4 (13.8)
Headache, *n* (%)	0	1 (7.1)	1 (3.4)
Lower gastrointestinal symptoms (e.g., obstipation), *n* (%)	2 (13.3)	1 (7.1)	3 (10.3)
Voiding disorders, *n* (%)	3 (20)	1 (7.1)	4 (13.8)
Attentive disorders, *n* (%)	3 (20)	1 (7.1)	4 (13.8)
Sensory disorders, *n* (%)	1 (6.7)	2 (14.3)	3 (10.3)
Fatigue, *n* (%)	5 (33.3)	1 (7.1)	6 (20.7)
Disease duration at the start of the study (years), mean ± SD (median)	3.9 ± 3.5 (3)	4.7 ± 4.1 (4)	4.3 ± 3.8 (3)
Duration of motor symptoms at the start of the study (years), mean ± SD (median)	4.5 ± 3.8 (3)	5.6 ± 4.5 (5.5)	5 ± 4.1 (4)
l-Dopa equivalent dose (mg), mean ± SD (median), dropouts included	417.5 ± 272.9 (380)	460.6 ± 403.6 (332.5)	438.3 ± 336.7 (380)
l-Dopa equivalent dose (mg), mean ± SD (median) first visit, without dropouts	431 ± 267.3 (380)	431 ± 443.1 (300)	431 ± 365.9 (313)
l-Dopa equivalent dose (mg), mean ± SD (median) last visit, without dropouts	439 ± 297.4 (300)	488 ± 475.6 (353)	464 ± 397.4 (300)
Systolic blood pressure value supine (mmHg), mean ± SD (median)	144.5 ± 30.1 (143.1)	134.1 ± 19.6 (126.7)	139.5 ± 25.7 (127.5)
Diastolic blood pressure value supine (mmHg), mean ± SD (median)	71.1 ± 12.3 (66.7)	66.3 ± 9.0 (65.0)	68.8 ± 10.9 (65.7)
Systolic blood pressure value upright (mmHg), mean ± SD (median)	121.4 ± 32.8 (119.5)	125.0 ± 24.1 (115.5	123.1 ± 28.5 (118.0)
Diastolic blood pressure value upright (mmHg), mean ± SD (median)	65.4 ± 14.0 (63.4)	67.3 ± 12.5 (68.1)	66.3 ± 13.1 (65.7)
Initial NOH, *n* (%)	1 (6.7)	1 (7.1)	2 (6.9)
Classical NOH, *n* (%)	7 (46.7)	6 (42.9)	13 (44.8)
Delayed NOH, *n* (%)	8 (53.3)	8 (57.1)	16 (55.2)
Maximal systolic change (mmHg), mean ± SD (median)	− 54.5 ± 28.4 (− 45.2)	− 46.2 ± 13.2 (− 45.5)	− 50.5 ± 22.4 (− 45.2)
Maximal diastolic change (mmHg), mean ± SD (median)	− 23.1 ± 14.7 (− 19.3)	− 21.4 ± 10.4 (− 21.4)	− 22.3 ± 12.6 (− 19.3)
Maximal heart rate increase (beats/min), mean ± SD (median)	19.9 ± 11.3 (17.7)	20.2 ± 14.1 (17.0)	20.0 ± 12.5 (17.7)
Mean systolic change (mmHg), mean ± SD (median)	− 23.1 ± 28.0 (− 15.5)	− 9.1 ± 20.4 (− 11.3)	− 16.3 ± 25.3 (− 14.1)
Mean diastolic change (mmHg), mean ± SD (median)	− 5.7 ± 12.8 (− 3.2)	1.0 ± 8.0 (2.9)	− 2.5 ± 11.1 (− .3)
Mean heart rate increase (beats/min), mean ± SD (median)	6.2 ± 8.0 (5.4)	6.4 ± 5.2 (5.7)	6.3 ± 6.7 (5.4)
Time to reach systolic minimum (includes 5-min laying time) (min), mean ± SD (median)	8.7 ± 3.0 (7.9)	7.5 ± 2.8 (6.1)	8.1 ± 2.8 (6.8)
Time to reach diastolic minimum (includes 5-min laying time) (min), mean ± SD (median)	9.7 ± 3.1 (9.0)	8.3 ± 3.6 (6.7)	9.0 ± 3.4 (8.7)
Time to reach maximal heart rate (includes 5-min laying time) (min), mean ± SD (median)	9.6 ± 3.7 (9.1)	10.2 ± 5.9 (8.7)	9.9 ± 4.8 (8.9)
RSA (diff. RRmax-RRmin) (msec), mean ± SD (median)	144.8 ± 174.7 (96)	80.4 ± 94.8 (40)	113.7 ± 143.2 (56)
Valsalva, mean ± SD (median)	1.5 ± 0.4 (1.3)	1.4 ± 0.5 (1.2)	1.5 ± 0.5 (1.3)
Ewing: 30:15-Quotient, mean ± SD (median)	1.5 ± 0.8 (1.1)	1.3 ± 0.7 (1.1)	1.4 ± 0.8 (1.1)
2-min walking test (m), mean ± SD (median)	154.0 ± 38.4 (155)	161.0 ± 48.2 (180)	157.3 ± 42.5 (161)
Winker, median ± IQR	19 ± 8	10 ± 15	17 ± 14
BDI-II, median ± IQR	13 ± 11	9 ± 13	12 ± 11
ESS, median ± IQR	11 ± 12	8.5 ± 10	10 ± 10
PDQ-39, median ± IQR	59 ± 25	39 ± 35	46 ± 33
NMS, median ± IQR	16 ± 8	10.5 ± 7	12.5 ± 7
UPDRS I, median ± IQR	3.5 ± 1	2 ± 2	3 ± 2
UPDRS II, median ± IQR	14 ± 10	11 ± 9	12.5 ± 9
UPDRS III, median ± IQR	21.5 ± 17	22 ± 16	21.5 ± 18
Hoehn and Yahr, median ± IQR	2.5 ± 2	2.5 ± 4	2.5 ± 2
MoCA, median ± IQR	25 ± 6	26 ± 5	25.5 ± 5

Metric data are shown as the mean ± standard deviation with (median), ordinal data as median ± interquartile range and nominal data as the number of patients *n* (%)

*l-Dopa* levodopa, *mg* milligram, *mmHg* mm of mercury, *min*. minute, *m* meters, *msec* milliseconds, *RSA* respiratory sinus arrhythmia (diff. RRmax-RRmin. difference of maximal and minimal beat-to-beat-difference), *Valsalva* Valsalva quotient (RR maximum/RRminimum), *Winker* Winker’s scale score, *BDI-II* Beck’s depression inventory score, *ESS* Epworth sleepiness scale score, *PDQ-39* Parkinson disease questionnaire score, *NMS* Nonmotor symptoms questionnaire, *UPDRS* Unified Parkinson’s disease rating scale score, *MoCA* Montreal cognitive assessment (total score, with education correction)

## Discussion

This randomized controlled study powered to improve “maximal systolic blood pressure change” by approximately 10 mmHg with 8 weeks of leg muscle training failed to confirm an additional effect of strength training compared with symptomatic therapy alone. Nonetheless, the “maximal systolic blood pressure change” appeared to be lower after strength training.

Physiotherapeutic strategies for treating OH in PD are scarce. Most randomized controlled trials reported improvement in motor symptoms, quality of life, and an increase in muscle strength after a period of strength training in PD patients [6–8]. Previous experience with strength training and its effects on OH were inconclusive [6–8]. In particular, a study by Kanegusuku et al. [7] included 30 PF patients without neurogenic OH and reported positive effects on orthostatic stress response by a 12-week progressive strength training program. Although the intervention of the study appears to resemble ours, we did not find significance in our sample of patients with OH. Possible reasons for these conflicting results might be a longer training time in their study, as also the fact that our patients already suffered from OH [7]. In our study design, the expected effect of strength training on orthostatic blood pressure decline may have been overestimated. During a head-up tilt test, there is no activation of the muscle pump, which was targeted by the leg-muscle training. Thus, an active standing test would have been more appropriate to investigate the effects of lower extremity strength training on “maximal systolic blood pressure change” after standing. Future studies should be powered to explore the effect of strength training on other parameters such as orthostatic complaints and cognitive symptoms associated with PD. Progressive strength training of longer duration might be more effective than 8-week strength training. A limiting factor of this analysis is the missing data of muscle strength measurements to demonstrate a significant increase in muscle mass by the intervention.

Due to the comorbidities of the older PD patients, other disorders as polyneuropathy, diabetes, or arterial hypertension influencing the autonomic nervous system besides PD could not be ruled out. A possible residual effect of pressor agents or antihypertensive drugs on the tilt-table results could not be ruled out because patients took their last medication on the evening before the testing.

As this study has a small sample size and prospective registrations did not succeed, it has the character of a preliminary study with limited scientific findings.

## Conclusions

Targeted leg muscle strength training did not significantly improve orthostatic “maximal systolic blood pressure change” during head-up tilt in this preliminary study. Further controlled clinical trials with larger sample sizes and sufficient power are required to investigate the effect of strength training on OH and cognition in PD.

## Supplementary Information

Below is the link to the electronic supplementary material.

Supplementary file1 (TIF 640 KB)

Supplementary file2 (TIF 1074 KB)
