# Supplementary figures and images for: Effect of strength training on orthostatic hypotension in Parkinson’s disease—a pilot study
Source: Clin Auton Res. 2022 Jun 15;32(3):213–7. doi: 10.1007/s10286-022-00870-5 (PMC9236997; doi:10.1007/s10286-022-00870-5)

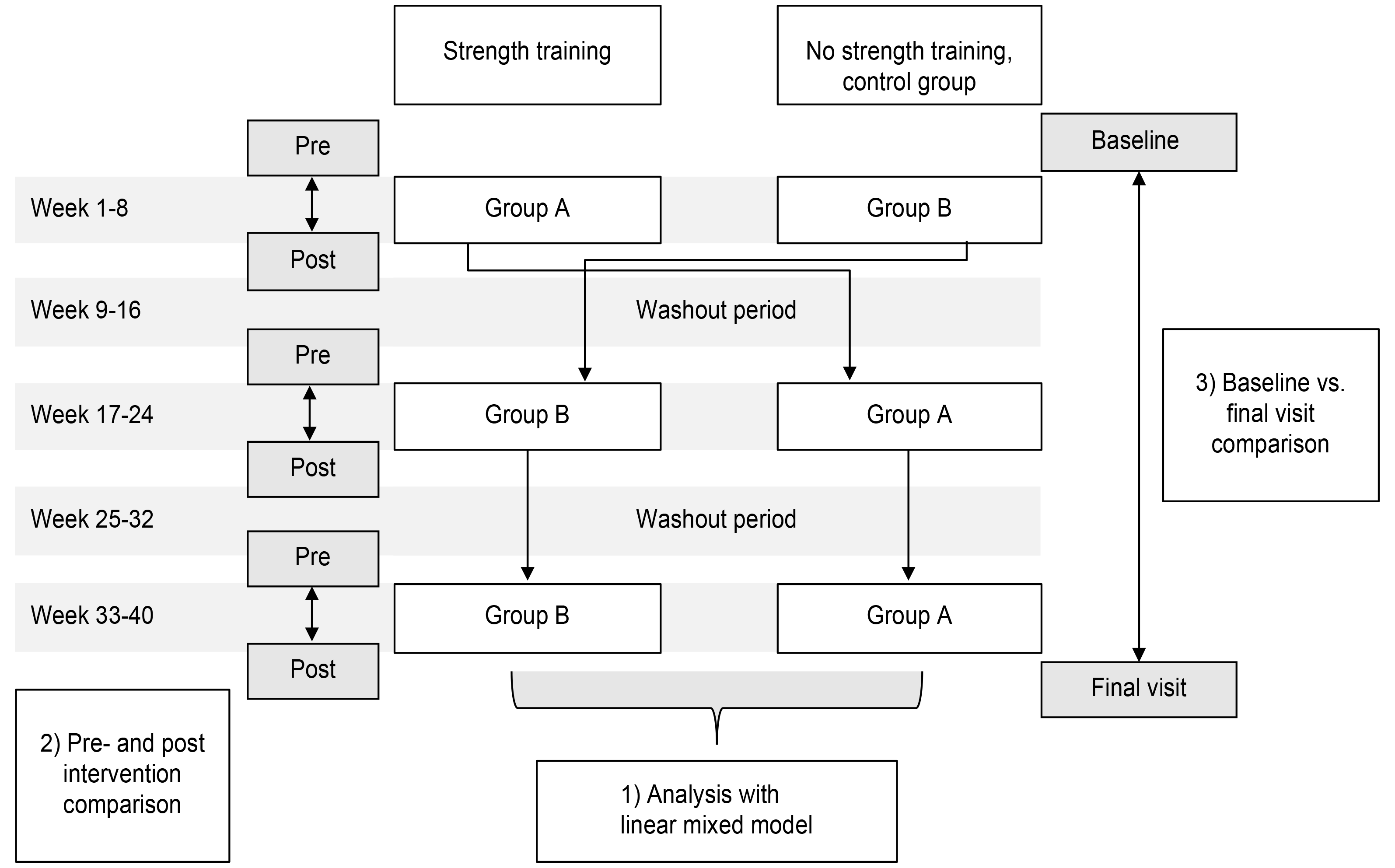

Supplement: Supplementary file 2 — Supplementary file2 (TIF 1074 KB) [file 10286_2022_870_MOESM2_ESM.tif]
